# Characterization of the nonlinear optical properties of nanocrystals by Hyper Rayleigh Scattering

**DOI:** 10.1186/1477-3155-11-S1-S8

**Published:** 2013-12-10

**Authors:** Cécile Joulaud, Yannick Mugnier, Gnon Djanta, Marc Dubled, Jean-Christophe Marty, Christine Galez, Jean-Pierre Wolf, Luigi Bonacina, Ronan Le Dantec

**Affiliations:** 1 Université de Savoie, SYMME, BP 80439, 74944 Annecy-le-Vieux Cedex, France; 2 GAP-biophotonics, Université de Genève, 22 chemin de Pinchat, 1211 Genève 4, Switzerland

## Abstract

**Background:**

Harmonic Nanoparticles are a new family of exogenous markers for multiphoton imaging exerting optical contrast by second harmonic (SH) generation. In this tutorial, we present the application of Hyper-Rayleigh Scattering (HRS) for a quantitative assessment of the nonlinear optical properties of these particles and discuss the underlying theory and some crucial experimental aspects.

**Methods:**

The second harmonic properties of BaTiO_3_, KNbO_3_, KiTiOPO_4 _(KTP), LiNbO_3 _and ZnO nanocrystals (NCs) are investigated by HRS measurements after careful preparation and characterization of colloidal suspensions.

**Results:**

A detailed analysis of the experimental results is presented with emphasis on the theoretical background and on the influence of some experimental parameters including the accurate determination of the nanocrystal size and concentration. The SH generation efficiency and averaged nonlinear optical coefficients are then derived and compared for six different types of NCs.

**Conclusions:**

After preparation of colloidal NC suspensions and careful examination of their size, concentration and possible aggregation state, HRS appears as a valuable tool to quantitatively assess the SH efficiency of noncentrosymmetric NCs. All the investigated nanomaterials show high SH conversion efficiencies, demonstrating a good potential for bio-labelling applications.

## Background

Imaging techniques are increasingly accompanied by nanotechnological developments aiming at the reliable, scalable, and efficient production of nanoparticle-based bio-markers with specific physical, optical, and chemical properties [1]. The use of multiphoton microscopy combined with fluorescent labels has undoubtedly led to improvements in terms of spatial resolution, depth penetration in biological tissues, and reduction of photo-damage [2,3]. However, these optical labels generally suffer from bleaching and blinking limiting optical contrast and observation time. A new generation of nanoparticles based on nonlinear optical response is being developed in this context to overcome these limitations and complement fluorescent labels. Second Harmonic emission from non-centrosymmetric nanocrystals (NCs) indeed presents appealing features for bio-imaging such as a long-term photostability and virtually unlimited wavelength flexibility[4-9].

In linear optics, we define the macroscopic polarization *P *induced on a medium by an electromagnetic field *E *(characterized by an oscillation frequency  ω or a wavelength λ=2πc/ω) as P∝χE. According to this relationship, the polarization is proportional to the incident field and the linear response of the medium is characterized by its susceptibility  χ. The radiated intensity is proportional to the incident intensity and the frequency remains the same. In nonlinear optics, the intensity of the incident electric field being much higher, the induced polarization is no longer proportional to the incident field and additional nonlinear terms must be included, $\propto \chi E+\chi^{\left(2\right)}E^{2}+\chi^{\left(3\right)}E^{3}+\ldots$ , where higher-order susceptibilities $\chi^{\left(2\right)}$, $\chi^{\left(3\right)}$ are tensors referring to the medium characteristics which determine its nonlinear optical response. SH generation is a second-order optical process depending on $\chi^{\left(2\right)}$ where a fraction of the incident light wave is converted into radiations with frequency twice the incident one. It is worth noting that bulk SH generation is only observed in noncentrosymmetric media, limiting the choice of harmonic nanoparticles to a specific class of materials.

Optical contrast based on frequency doubling process can bring several benefits for bio-imaging. First of all, involving exclusively virtual electronic states, it does not imply energy absorption preventing bleaching usually observed with fluorescent and luminescent nanoparticles. Therefore, it is possible to achieve observations over long time durations with no decrease in signal quality [10]. In addition, SH generation is a non-resonant process which can occur for any excitation wavelength [10-12]. Consequently, excitation wavelengths can be selected in spectral ranges where the biological tissue absorption and scattering are low so that photo-damage is limited and penetration depth increased [13]. A wise choice of the excitation wavelength also allows avoiding hindrance by sample autofluorescence increasing image contrast. In terms of emitted intensity, the SH signal scattered by NCs is related to the square intensity of incident power. Detection and imaging of such individual bio-markers generally requires high peak-power excitation beams, a condition nowadays easy achievable with femtosecond lasers even for very moderate pulse energies [13,14]. Practically, the preparation of SH nanocrystals as bio-labels involves a good knowledge in chemical synthesis and functionalization. Nanocrystals in the 10-100 nm size range with good crystallinity [15] and low cytotoxicity [10,16,17] are typically required as well as the development of conjugation techniques [18-20] for an efficient interaction between the NCs and the biomolecules of interest. Finally, the selection of optical labels with high SH efficiency requires the development of experimental techniques to quantitatively assess the nonlinear optical response of nanocrystals. SH generation is indeed well known in bulk crystals and can also be easily characterized in thin films and agglomerated powders. However, characterization techniques for nonlinear nanocrystal ensembles are sorely lacking. In this paper, we propose to apply the Hyper-Rayleigh Scattering (HRS) technique, originally developed for the investigation of harmonic light scattering in molecular solutions, in order to characterize the SH efficiency of solvent-dispersed non-centrosymmetric NCs.

The theory and first experimental detection of Hyper-Rayleigh Scattering were first exposed in 1965 [21,22]. In a series of experiments, HRS was later used to determine the second-order nonlinear optical properties of molecules including species with no permanent dipole moments [23,24]. The HRS intensity is defined as the incoherent sum of SH signals scattered by different sources excited by an incident field of intensity $I_{\omega }$: $I_{HRS}=G\sum_{i}\left(N_{i}F_{i}<\beta_{i}^{2}>\right)I_{\omega }^{2}$ , where the subscript *i *refers to the different species in solution. <β> stands for $\sqrt{<\beta^{2}>}$ which is the average amplitude of the second-order molecular hyperpolarizability and brackets indicate isotropic orientational averaging. This quantity describes the molecular nonlinear optical response considering that local microscopic fields E_loc _induce dipole moments $p_{2\omega }$ that can be written as $p_{2\omega }=\beta E_{loc}^{2}$ where *β *is here a third-rank tensor. *N_i _*is the species concentration, *F *a local field factor depending on the surrounding medium refractive index, and *G *an experimental proportionality constant. From the above HRS intensity equation, the square dependence on the incident intensity and the linear behaviour with the concentration species can be noticed. Besides, quantitative assessment of the hyperpolarizability <β> of nonlinear species with the external reference method is specifically based on this linear trend [23,24]. By varying the molecule concentration (N_i_) and measuring the corresponding HRS intensity (I_HRS_), one can obtain the slope α defined as $\alpha =GF<\beta^{2}>I_{\omega }^{2}$ from the $I_{HRS}\left(N_{i}\right)$ curve $\alpha =GF<\beta^{2}>I_{\omega }^{2}$. Hyperpolarizability is then derived by comparison with the experimental slope measured from a reference solution containing molecules of known hyperpolarizability. The above procedure assumes that the parameter *G *remains unchanged for defined experimental conditions whereas local field factors can be calculated from solvent indices.

## Methods

### Second harmonic NC characterization from HRS

HRS has been recently extended to larger objects such as molecular assemblies or nanometric- to microscopic-scale particles [25-31]. A delicate issue about nanocrystal characterization is that the microscopic and macroscopic description of the SH optical process seems inappropriate. As previously mentioned, HRS is usually employed to determine the molecular hyperpolarizability <β>. On the other hand, SH properties of bulk crystals are commonly associated with the macroscopic second order nonlinear susceptibility $\chi^{\left(2\right)}$ (note that $\chi^{\left(2\right)}=2d$, where *d *is the nonlinear optical coefficient in the convention I used here [32]). An effective hyperpolarizability can be therefore conveniently introduced with the purpose of relating microscopic to macroscopic parameters allowing a straightforward comparison with the bulk nonlinear optical properties. Assuming that NCs are large enough to neglect surface contribution of the scattered SH signal, the effective hyperpolarizability $<\beta_{nc}>$ has the same properties as $\chi^{\left(2\right)}$ and is related to the averaged SH coefficient <*d>*by the mean particle volume V_nc _[30,33] :

(1) $$<\beta_{nc}>=<d>V_{nc}$$

It should be mentioned here that, contrary to molecular hyperpolarizability, $<\beta_{nc}>$ is not defined according to local microscopic fields. An internal field factor *T *must however be introduced to better estimate the incident optical macroscopic field in the nanocrystal. In the case of colloidal suspensions of nanocrystals, there is indeed a refractive index difference between the solvent and the investigated nanomaterial. According to this definition, the HRS intensity of a given suspension containing NCs (subscript "nc") dispersed in a solvent (subscript "s") can be expressed as:

(2) $$I_{HRS}=G(N_{s}F_{s}<\beta_{s}^{2}>+N_{nc}T_{nc}<\beta_{nc}^{2}>)I_{\omega }^{2}$$

The external reference method can again be applied to determine $<\beta_{nc}>$ provided that the suspension concentration is known. In addition, quantitative assessment of the <d> parameter also assumes that the nanocrystal size is independently estimated.

Our HRS experimental set-up [34] is based on a Q-switched Nd:YAG laser (Wedge HB, Bright solutions, 1 mJ/1 ns). The vertically polarized beam is focused by a 20 cm focal length lens into a glass cuvette containing the NCs suspension. Scattered SH signal is collected at 90° from the laser beam axis with a 5 cm focal length lens and measured by a photomultiplier tube. A colored glass filter (short pass) and an interferometric filter (532 nm) are placed before the photomultiplier to remove unwanted signals. The unpolarized HRS signal is finally processed by a boxcar amplifier after integration and averaging over typically 1000 laser shots.

### Preparation of NC suspensions

Different types of particles are investigated in this work: agglomerated BaTiO_3 _and KNbO_3 _nanopowders obtained by ball grinding of bulk crystals (kindly supplied by FEE GmbH, Idar-Oberstein, Germany), KTP nanocrystals extracted from raw powder at the end of the flux-growth process (kindly provided by Cristal Laser, Messein, France) [35]. LiNbO_3 _nanocrystals synthesised by a hydrolysis method [36] supplied by the SRSMC laboratory (Nancy, France), and, two commercial batches of ZnO with different average nominal sizes of 20 nm and 90-200 nm purchased from NanoAmor Inc. (Houston, USA).

Colloidal suspensions with low size dispersion and good stability are obtained following a specified protocol [10,37]. Dry nanopowders are first dispersed in water or ethanol at 0.5 mg/mL concentration and sonicated (Vibra Cell 75043, Bioblock Scientific) during 25 min in pulsed mode (1 s on, 4 s off). Large particles and remaining aggregates are then excluded by leaving the solution settling down during a period of 1 to 7 days depending on the NCs type. As-obtained supernatants have a typical concentration of 0.1 mg/mL. Size and size dispersion are then characterized by Dynamic Light Scattering (Malvern ZetasizerNanoZS). Concentration of solvent-dispersed NCs is measured after evaporation of a precise volume of the obtained suspension and weighing the residual.

## Results

### Hyper-Rayleigh Scattering Data Analysis

AHRS intensity measurements are performed according to the concentration of NCs and, on the other hand, of para-nitroaniline (pNA) diluted in methanol which is used here as the reference molecular solution. After linear fitting of the experimental coefficients $\alpha_{NC}$ and $\alpha_{pNA}$ (that relate I_HRS _to the nanocrystal and pNA concentrations, respectively), the effective hyperpolarizability $<\beta_{nc}>$ is calculated as [30,33]:

(3) $$<\beta_{nc}>\;=\sqrt{\frac{\alpha_{nc}F_{pNA}}{\alpha_{pNA}T_{nc}}}<\beta_{pNA}>$$

$F_{pNA}$, the Lorentz-Lorenz local field factor correction, is given by $F_{pNA}={\left(\frac{n_{meth}^{2}+2}{3}\right)}^{6}$ with $n_{meth}$ the refractive index of methanol. The internal field factor is expressed as $T_{nc}={\left(\frac{3n_{s}^{2}}{2n_{s}^{2}+n_{nc}^{2}}\right)}^{6}$ where $n_{s}$ and $n_{NC}$ are the refractive indices (considered here without index dispersion *i.e*., n(ω)~n(2ω)) of solvent and NCs, respectively. The reference value of the pNA molecular hyperpolarizability is $\beta_{33}=25.9\times 10^{-30}esu$[38] from which the orientationally averaged hyperpolarisability corresponds to $\sqrt{\frac{6}{35}\beta_{33}}$ in our experimental configuration (vertical input polarization and no analyser).

To allow a comparison with bulk crystal literature values, the averaged nonlinear coefficient <d> is then obtained according to equation (1) by inserting the volume $V_{nc}$. This procedure applied to the 6 available types of NCs, leads to the experimental determination of the effective hyperpolarizabilities and averaged nonlinear coefficients as shown in table 1. The validity of the presented protocol is discussed hereafter with a special focus on the possible optical artefacts associated with the SH measurements. Experimental precautions and recommendations are also introduced with regard to the influence of the size, size distribution and aggregation state of the solvent-dispersed NCs.

**Table 1:** Results

	Mean DLS size D_m _[nm]	$<\beta_{nc}>$**[10^-24 ^esu]**	<d_mono_> [pm/V]	<d_poly_> [pm/V]	<d_bulk_>* [pm/V]
BaTiO_3_	91	5.16	5.5		14.1 - [45]

KNbO_3_	128	21.8	8.3	5.6	15.3 - [46]

KTP	237	33.5	2.8	1.4	7.6 - [46]

LiNbO_3_	125	16.3	7.4	4.9	17.3 - [47]

ZnO 90-200	132	7.12	2.5	1.2	2.8 - [48]

ZnO 20	50	0.33	2.1		2.8 - [48]

Mean NC size considering DLS distribution by number, effective hyperpolarizability $<\beta_{nc}>$, averaged nonlinear optical coefficient derived from monodisperse ($<d_{mono}>$) and polydisperse ($<d_{poly}>$) suspensions and comparison with literature values ($<d_{bulk}>$) for the different SH nanocrystals. Error bars for $<\beta_{nc}>$ and $<d_{mono}>$ are estimated at ± 15% in terms of experimental reproducibility.

*<d^2^> = 6/35 d_33_^2 ^+ 32/105 d_31_.d_33 _+ 92/105 d_31_^2 ^for BaTiO_3 _(4 mm) and ZnO (6 mm)

<d^2^> = 6/35 d_33_^2 ^+ 38/105 (d_32_^2 ^+ d_31_^2^) + 16/105 (d_31_.d_32 _+ d_31_.d_33 _+ d_32_.d_33_) for KTP (mm2) and KNbO_3 _(mm2)

<d^2^> = 6/35 d_33_^2 ^+ 92/105 d_31_^2 ^+ 8/21 d_22_^2 ^+ 32/105 d_31_.d_33 _for LiNbO_3 _(3 m)

### Optical considerations for a correct HRS measurement

**Figure 1:**
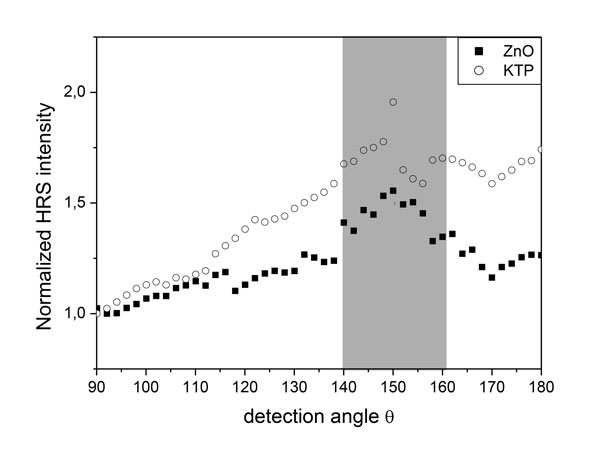
**Rayleigh**. Variation of the HRS intensity of ZnO and KTP suspensions for a collection angle spanning from 90° to 180° with respect to the incident beam direction. Strong fluctuations observed between 140° and 160° are attributed to a non-significant interaction of the incident laser beam with optical components the rotating detection arm.

Among the optical considerations and possible artefacts, let us first point out that the term HRS is specifically used here because scattering at 2ω is detected with the classical HRS experimental configuration. According to the recent review of Roke *et al*. [31] Second Harmonic Scattering would be more appropriate for ~100 nm non-centrosymmetric NCs. If an incoherent process is underlying in eq.2 (I_HRS _increases linearly with the NC concentration), the SH intensity also depends quadratically on the NC volume as it can be seen after combination of eq.1 with eq.2. The number of induced nonlinear dipoles (at the scale of the crystalline unit cell) being proportional to V_nc_, a coherent process thus takes place within each NC between the various radiated SH fields. This bulk response is equivalent to the one observed in single crystals except that phase matching constraints are absent because the NC size is well below the coherent length [5]. Coherent effects are thus to be considered within each NC whereas the incoherent contribution originates from the linear concentration dependence. The Rayleigh regime assumption, at the very basis of HRS theory could be verified for most NC suspensions. The scatterers are assumed to be point-like, *i.e*. much smaller than the excitation wavelength so that light is scattered isotropically. An experimental verification can be carried out by detecting the normalized HRS intensity of a NC suspension at different collection angles spanning from 90° to 180° with respect to the incident beam direction. Figure 1 shows the curves obtained for ZnO and KTP. The ZnO suspension (130 nm average particle diameter) displays an almost flat angular response leading to conclude on the validity of the Rayleigh regime. The KTP suspension, on the other hand, containing larger NCs of around 200 nm presents higher forward scattering of I_HRS _evidencing the limit of the Rayleigh regime assumption in this size range.

**Figure 2:**
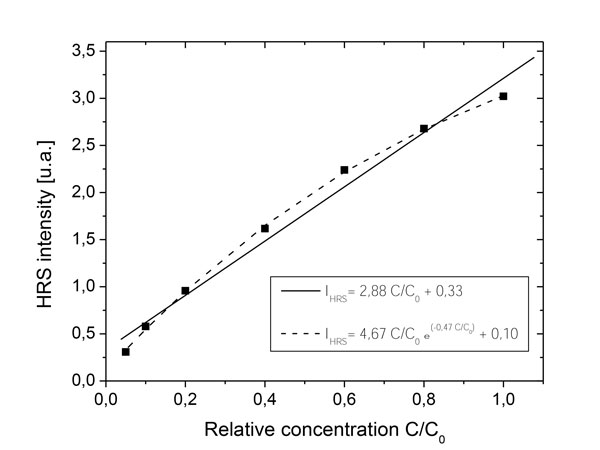
**Scattering**. Deviation from the expected linear HRS response in the case of a highly concentrated KNbO_3 _suspension.

In regard to the possible optical artefacts, only the nonlinear elastic scattering is to be measured and a monochromator is necessary to spectrally resolve the HRS signal and to detect the presence of any possible inelastic process. This basic verification has been done for all the NCs under consideration. We only observed second harmonic scattering and no fluorescence [39]. Light absorption and scattering can also lead to alteration of the HRS signal. For instance, light absorption at the working wavelengths (1064 and 532 nm) tends to reduce the SH intensity when the species concentration is too high. This results in a deviation from the linear HRS response and a Beer-Lambert correction factor is to be added to correctly estimate the expected proportionality between the HRS intensity and the concentration [40]. Similarly, a deviation has also been observed with non-absorbing NCs (see Figure 2) so that an exponential correction was introduced to account for the very high multiscattering processes of the incident and SH radiations. Note that further dilution of the initial supernatants readily allows the recovery of a linear dependence on the NC concentration.

### Influence of the NC size, concentration and possible aggregation state

Size characterization of NCs is of paramount importance for the correct determination of SH efficiencies. As previously described, the averaged nonlinear optical coefficient <d> is retrieved from $<\beta_{nc}>$ and the mean NC volume. Combination of eq.1 with eq.2 shows a dependence of HRS intensity with the squared NCs volume $V_{nc}^{2}$, emphasizing the requirement of a precise size characterization. In addition, assessment of the suspension concentration also relies on the NC size. As detailed hereafter, a mass concentration is indeed first measured and the unit particle concentration then estimated according to the NC volume.

**Figure 3:**
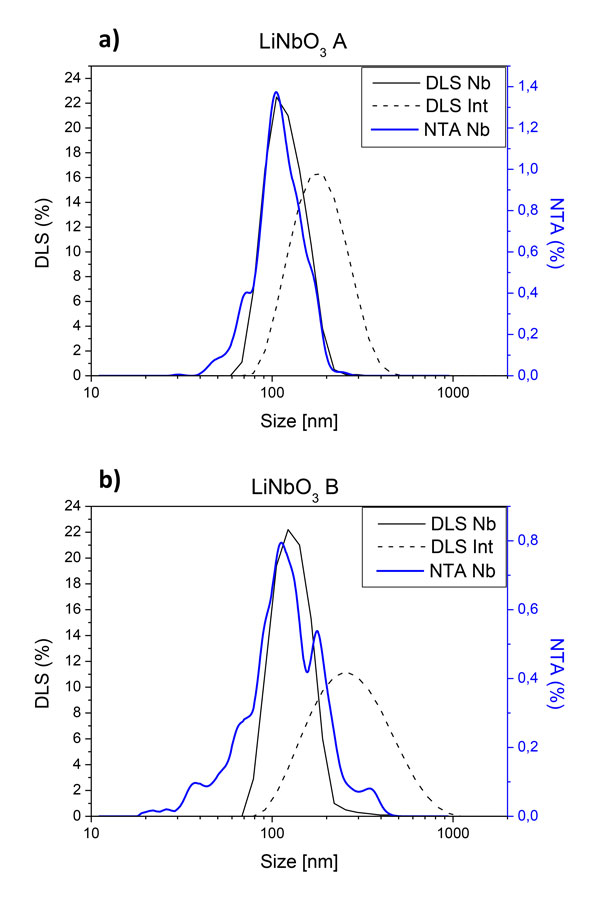
**Size**. DLS size distributions by intensity and number for two different suspensions A (a) and B (b) of LiNbO_3 _NCs. Comparison is made with the NTA distribution by number.

Size characterization can be performed using Dynamic Light scattering (DLS), a technique based on the intensity fluctuations in the scattered light. These fluctuations are caused by Brownian motion and can be related to the particle size. This method provides a particle size distribution in intensity since the detected signal is proportional to a linear elastic scattering. A main issue may arise from size polydispersity because large particles can readily hinder the lower intensity response of smaller scatterers. In Figure 3, two different LiNbO_3 _suspensions are characterized by DLS. Sample A was prepared as described in "methods" whereas the sedimentation step was not completed for sample B. A comparison is made with the distributions obtained from a particle-by-particle investigation technique called Nanoparticle Tracking Analysis (NTA) [41]. The good agreement between both results for sample A demonstrates the reliability of DLS measurements in that case. On the contrary, the larger dispersion associated with suspension B results in a less consistent correlation. This again points out the importance of paying a particular attention to suspension preparation.

Other critical issues complicating the interpretation of DLS results are related to the nanoparticles shape and the presence of aggregates. DLS indeed detects the hydrodynamic volume of objects and uses spherical models making the size characterization less relevant for non-spherical particles. Complementary observations of NCs by electron microscopy may help to obtain more information about the NC shape polydispersity. In particular, the discrimination between eventual aggregates and large individual NCs is not possible with DLS. As an exemplary result, ageing of the initial water-based solutions may result in a slow aggregation if electro-steric repulsions are too weak after the initial preparation of NC suspensions. From our experience, the long term stability of water-dispersed NCs is particularly difficult to achieve with BaTiO_3_, ZnO and KTP NCs and the use of ethanol as a solvent is generally preferred.

**Figure 4:**
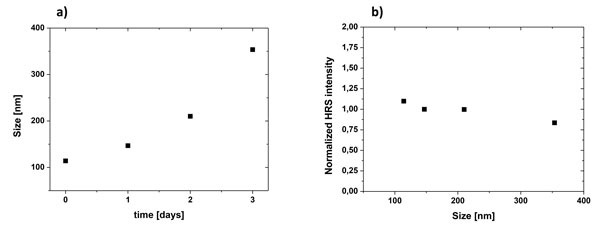
**Aggregation**. Effect of the aggregation state of ZnO NCs on the measured HRS intensity: a) time-dependent DLS measurements showing the apparent aggregate size, b) HRS intensity evolution with aggregates size.

Influence of the aggregation state on the HRS signal is specifically investigated in Figure 4. Simultaneous DLS and HRS measurements for ZnO NCs suspended in ethanol have been carried out for several days. The size increase in DLS measurements is typical of a spontaneous aggregation process (Figure 4a) whereas the almost constant HRS signal attests of the low effect of aggregation on the SH intensity (Figure 4b). This experiment unambiguously demonstrates that the HRS signal only depends on the individual nanocrystal size. Even after aggregation, the incoherent contribution of each individual nanocrystal is preserved indicating the absence of specific orientations within aggregates. On the contrary, the individual orientation and dynamics of spontaneous aggregation were observed to be well different for BaTiO_3 _NCs previously dispersed in a nonpolar fluid, like heptane [42,43].

Finally, accuracy of concentration measurements should also be clarified. In our experience, the weighting method used to determine the mass concentration is satisfactory provided that the evaporated volume is sufficient. Typically, a 30 mL suspension is evaporated in several successive steps in light containers of 7 g. The resulting relative uncertainties in the mass concentration are between 1 and 5%. $N_{nc}$, the unit particle concentration, is then calculated from the mass concentration $C_{m}$ with $N_{nc}=\frac{C_{m}}{\rho V_{NC}}$ where  ρ is the material density and $V_{nc}$ the mean NC volume. As individual NCs are monitored with the NTA analysis (for which the preparation of very diluted suspensions is a prerequisite), comparison between the two approaches is straightforward. Deviations by a factor of 1.5 were noticed in the best cases, which is acceptable considering the inherent size polydispersity that affects the accuracy of the weighing procedure.

### Influence of the NC size distribution

Another point of interest is that the sizes used for derivation of the nonlinear optical parameters are actually the mean values of distributions. In other words, HRS intensity is considered as a nonlinear elastic scattering from exactly monodisperse samples. Because of the already mentioned size polydispersity, the HRS signal (that depends quadratically on V_nc_) indicates that a mean value is not commensurate with the real scattered SH intensity. In order to evaluate the influence of the actual size distribution width, we have compared the HRS intensities of a virtual (or ideal) monodisperse suspension to an actual polydisperse one. To begin, we only take the volume into consideration. We also assume that both distributions of spherical NCs (with diameter D) have the same mean size, $D_{m}$, but a non-zero standard deviation for the actual suspension. To that end, DLS distributions by number are fitted by using a normal probability density function ($\rho \left(D\right)$). Ratios of the HRS intensities between polydisperse and monodisperse samples can be then calculated as: $\frac{I_{poly}}{I_{mono}}=\frac{1}{D_{m}^{6}}\int D^{6}\rho \left(D\right)dD$. In the case of the LiNbO_3 _suspension A of Figure 3a, the calculated HRS intensity (with a 125 nm mean diameter and a 25 nm standard deviation) is for instance twice higher than a monodisperse suspension containing exclusively 125 nm NCs.

The statistical approach being introduced, our final aim is to evaluate the effect of the NC size distribution on the derived averaged nonlinear optical coefficient <d>. Concentration (N_nc_) and volume (V_nc_) are thus to be considered. Combination of eq.1 with eq.2 allows to write $<d^{2}>\propto I_{2\omega }/\left(N_{nc}\cdot V_{nc}^{2}\right)$. As discussed above for the concentration determination, $N_{nc}$ is inversely proportional to the NC volume so that the effect of a size-dispersion on the calculated concentration can be obtained from $\frac{N_{poly}}{N_{mono}}=\frac{D_{m}^{3}}{\int D^{3}\rho \left(D\right)dD}$. Taking into account the squared volume, the final effect of a size dispersion for spherical NCs with diameter D on the averaged <d> coefficient can be expressed as: $\frac{<d_{poly}>}{<d_{mono}>}=\sqrt{D_{m}^{3}\frac{\int^{D^{6}}\rho \left(D\right)dD}{\int^{D^{3}}\rho \left(D\right)dD}}.$

Interestingly, the whole effect of the NC-size dispersion for the LiNbO_3 _A suspension is relatively weak. A factor of about 0.8 is indeed found between the "polydisperse" and the "monodisperse" averaged coefficient showing that <d> is generally overestimated when the size distribution is ignored.

Finally and after consideration of the previous recommendations and precautions, our experimental results for the 6 different NC type are summarized in table 1. The mean NC size (D_m_) is obtained from a DLS distribution by number whereas the effective hyperpolarizabilities $<\beta_{nc}>$ are derived from eq.3. The averaged nonlinear optical coefficient calculated with monodisperse $<d_{mono}>$ and polydisperse $<d_{poly}>$ (when available) suspensions are then compared with the literature values $<d_{bulk}>$ of the different material. Note that literal expressions of $<d_{bulk}>$ (corresponding to our experimental polarization configuration) can be found in footnote of table 1. They have been calculated assuming Kleinman conditions and the symmetry class of each material [24,44].

## Discussion

Among the different experimental optical parameters, let us remind that $<\beta_{nc}>$ is characteristic of the NC SH efficiency. The effective hyperpolarizabilities $<\beta_{nc}>$ indeed linearly depend on the NC volume whereas the averaged nonlinear optical coefficient <d> is an intensive physical property of each material. This distinction is readily observed with the two ZnO samples that display similar <d> coefficients confirming that the bulk contribution is predominant in the measured HRS signal. Consistently, large-size NCs (ZnO 90-200) have a higher hyperpolarizability than small NCs (ZnO 20). For KTP NCs, the highest measured effective hyperpolarizability of $33.5\times 10^{-24}$ esu is to be related to the relative large NC volume and not to especially efficient intrinsic properties.

In addition, it can be noticed that the <d> coefficients for the different materials are otherwise very similar (a few pm/V) and that the comparison with literature $<d_{bulk}>$ values obtained from bulk crystals is also very consistent. Such an agreement is in favour of the HRS technique as a quantitative characterization method and also confirms the absence of a significant surface contribution in the scattered SH intensity. On the other hand, if the apparent hierarchy between bulk properties of the different materials seems preserved, a systematic discrepancy can be noticed. Literature bulk crystal and experimental NCs <d> coefficients indeed differ by a factor 2 to 3 and several explanations can be suggested. Our quantitative assessment is first based on the external reference method with pNA molecules for which various different nominal hyperpolarizabilities have been published. Similarly, the literature nonlinear optical coefficients differ for bulk materials. For the NCs, the possible presence of impurities and amorphous phases has not been evidenced through X-Ray diffraction (data not shown) but slight deviations from the chemical stoichiometry could also alter the SH properties. Finally, it is our opinion that this systematic shift most probably results from the inherent size- and shape-distributions together with the likely presence of aggregates that inevitably leads to an underestimation of the nonlinear optical coefficient <d>. The likelihood of different ferroelectric domains in the investigated materials (except ZnO for which the experimental and literature are well consistent) also points out to the necessity to improve current strategies for producing NCs with accurate size, shape and composition.

## Conclusions

Application of the HRS technique on NC suspensions allows to achieve a reliable assessment of the SH efficiencies of noncentrosymmetric nanomaterials in the context of their promising application for bio-imaging as nonlinear optical markers. Limitations and experimental *caveats *of the method have been thoroughly discussed, with special attention to the crucial size and concentration issues. Our conclusions emphasize the importance of the preparation of aggregate-free NC suspensions with ideally monodisperse samples in terms of size and shape. These recommendations should rapidly improve accuracy of these quantitative measurements. To date, we can however assert that the different investigated NCs all present very similar nonlinear optical coefficients <d> and thus comparable SH efficiencies at a given NC size. A pertinent selection of a NC type for bio-imaging also weakly depends on the nonlinear optical properties. In the future, the reliable and scalable chemical synthesis of size- and shape-controlled NCs as well as their cytotoxicity and biocompatibility are the relevant parameters to consider.

## Competing interests

The authors declare that they have no competing interests.

## Authors' contributions

CJ, RLD, YM, LB wrote the paper. CJ, MD, RLD, YM, JCM performed the experiments. CG, YM, RLD designed the experimental set-up.
